# Supplementary material for: Validation of Two Screening Tools for Detecting Delirium in Older Patients in the Post-Anaesthetic Care Unit: A Diagnostic Test Accuracy Study
Source: Int J Environ Res Public Health. 2022 Nov 30;19(23):16020. doi: 10.3390/ijerph192316020 (PMC9738308; doi:10.3390/ijerph192316020)
Supplement: Supplementary file 1 [file ijerph-19-16020-s001.zip › ijerph-1976001-supplementary.pdf]

Supplementary Table S1. STARD checklist for reporting of diagnostic test accuracy studies

Section & Topic	No	Item	Reported on page #
TITLE OR ABSTRACT			
	1	Identification as a study of diagnostic accuracy using at least one measure of accuracy (such as sensitivity, specificity, predictive values, or AUC)	1
ABSTRACT			
	2	Structured summary of study design, methods, results, and conclusions (for specific guidance, see STARD for Abstracts)	1
INTRODUCTION			
	3	Scientific and clinical background, including the intended use and clinical role of the index test	2-3
	4	Study objectives and hypotheses	3
METHODS			
Study design	5	Whether data collection was planned before the index test and reference standard were performed (prospective study) or after (retrospective study)	3
Participants	6	Eligibility criteria	4
	7	On what basis potentially eligible participants were identified (such as symptoms, results from previous tests, inclusion in registry)	4-6
	8	Where and when potentially eligible participants were identified (setting, location and dates)	4-6
	9	Whether participants formed a consecutive, random or convenience series	7
Test methods	10a	Index test, in sufficient detail to allow replication	5-6
	10b	Reference standard, in sufficient detail to allow replication	5-6
	11	Rationale for choosing the reference standard (if alternatives exist)	7-8
	12a	Definition of and rationale for test positivity cut-offs or result categories of the index test, distinguishing pre-specified from exploratory	5-7
	12b	Definition of and rationale for test positivity cut-offs or result categories of the reference standard, distinguishing pre-specified from exploratory	5-7
	13a	Whether clinical information and reference standard results were available to the performers/readers of the index test	5-6

	13b	Whether clinical information and index test results were available<br>to the assessors of the reference standard	5-6
Analysis	14	Methods for estimating or comparing measures of diagnostic accuracy	8
	15	How indeterminate index test or reference standard results were handled	8
	16	How missing data on the index test and reference standard were handled	8
	17	Any analyses of variability in diagnostic accuracy, distinguishing pre-specified from exploratory	8
	18	Intended sample size and how it was determined	7-8
RESULTS			
Participants	19	Flow of participants, using a diagram	8
	20	Baseline demographic and clinical characteristics of participants	8-9
	21a	Distribution of severity of disease in those with the target condition	8-9
	21b	Distribution of alternative diagnoses in those without the target condition	8-9
	22	Time interval and any clinical interventions between index test and reference standard	10
Test results	23	Cross tabulation of the index test results (or their distribution) by the results of the reference standard	10-12
	24	Estimates of diagnostic accuracy and their precision (such as 95% confidence intervals)	10-12
	25	Any adverse events from performing the index test or the reference standard	10-12
DISCUSSION			
	26	Study limitations, including sources of potential bias, statistical uncertainty, and generalisability	13-15
	27	Implications for practice, including the intended use and clinical role of the index test	15-16
OTHER INFORMATION			
	28	Registration number and name of registry	18
	29	Where the full study protocol can be accessed	18
	30	Sources of funding and other support; role of funders	17

Supplementary Table S2: 3D-CAM Instrument

3D-CAM tool [CAM copyright 2003, Hospital Elder life, LLC Not to be reproduced without permission]						
Coding Instructions: Incorrect also includes "I don't know", and No response/ non-sensical response.<br>For any" Incorrect" or "Yes" responses, check the box in the final column designating which feature is present			CAM Feature			
Read: I have some questions about your thinking and memory…	Correct	Incorrect	1	2	3	4
1. Can you tell me the year we are in right now?						
2. Can you tell me the day of the week?						
3. Can you tell me what type of place is this? [hospital]						
4. I am going to read some numbers. I want you to repeat them in backwards order from the way I read them to you. For instant, if I say "5-2", you would say "2-5"<br>Ok? The first one is "7-5-1" (1-5-7).						
5. The second is "8-2-4-3" (3-4-2-8).						
6. Can you tell me the days of the week backwards, starting with Saturday?<br>(S, F, T, W, T, M, S), may prompt with "What is day before… for up to 2 prompts".						
7. Can you tell me the months of the year backwards, starting with December?<br>(D, N, O, S, A, J, J, A, M, F, J), may prompt with "What is month before… for up to 2 prompts".						
8. During the past day have you felt confused? about basic information (i.e., orientation, reason for hospitalisation) not details of medical condition/ treatment						
9. During the past day did you think that you were not really in the hospital?						
10. During the past day did you see things that were not really there?						
Observer Ratings: To be completed after asking patient questions 1-10.	No	Yes	1	2	3	4
11 a. Was the patient sleepy during the interview? (requires that they actually fall asleep but is easy to arouse)						
11 b. Was the patient stuporous, or comatose during the interview? Difficult to impossible to arouse						
12. Did the patient show hypervigilance, such as having strong responses to ordinary objects/ stimuli in the environment, being inappropriately startled, etc?						
13. Was the patients' flow of ideas unclear or illogical, for example tell a story unrelated to the interview (tangential)?						
14. Was the patients' conversation rambling, for example did he/she give inappropriately verbose and off target responses?						

15. Was the patients' speech unusually limited or sparse? (e.g., yes/no)						
16. Did the patient have trouble keeping track of what was being said in the interview?						
17. Did the patient appear inappropriately distracted by environmental stimuli?						
18. Did the patient's level of consciousness fluctuate during the interview, for example, start to respond appropriately and then drift off?						
19. Did the patient's level of attention fluctuate during the interview, e.g., did the patient's focus on the interview or performance on the attention tasks vary significantly?						
20. Did the patient's speech/ thinking fluctuate during the interview, for example, patient spoke slowly, then spoke very fast?						
Optional questions: Complete only if feature 1 is not checked and feature 2 is checked and either feature 3 or 4 is checked						
21. Consult the medical record or contact a family member, friend, or health care provider who knows the patient well to find out if the patient is experiencing an acute change. "Is the patient experiencing an acute change in their memory or thinking?"						
22. If second day of hospitalisation or later and previous 3D-CAM ratings are available: Review previous 3D-CAM assessments and determine if there an acute change in performance, based on any new: positive" items						
CAM Summary: Check if Feature present in column above			1	2	3	4
Delirium requires Feature 1 and 2 and either 3 or 4: -------------------<br>Present---------------------- Not Present						

Supplementary Table S3. DSM-5 criteria for delirium diagnosis

DSM-5 delirium Criteria/ exclusion	Yes	No	Don't know
A) A disturbance in;			
attention-reduced ability to direct, focus, sustain, and shift attention			
Awareness-reduced orientation to the environment			
B) The disturbance:			
Develops over a short period of time (usually hours to a few days)			
Represent a change from baseline attention and awareness			
Tends to fluctuate in severity during the course of the day			
C) An additional disturbance in cognition (e.g., memory deficit, disorientation, language, visuospatial ability, or perception).			
D) Exclusions-The disturbance in Criteria A and C are:			
Better explained by another pre-existing, established, or evolving neurocognitive disorder			
Occur in the context of a severely reduced level of arousal such as coma			
E) There is evidence from the history, physical examination or laboratory findings that the disturbance is a direct physiological consequence of another medical condition, substance intoxication or withdrawal (e.g., due to a drug abuse or to a medication), or exposure to a toxin, or due to multiple etiologies.			

Initial diagnosis:		Yes/ No
	Delirium-all items A, B, C and E = Yes, Plus D= No	
	No delirium-if any item in A, B, C and E=No, or D=Yes	
	Possible delirium- if any don't know in A, B, C and D, and No in E	
Revised Diagnosis: Date:	Delirium/ Possible delirium/ No delirium	

CC BY SA
